# Neuroprotection of Sanhua Decoction against Focal Cerebral Ischemia/Reperfusion Injury in Rats through a Mechanism Targeting Aquaporin 4

**DOI:** 10.1155/2015/584245

**Published:** 2015-05-18

**Authors:** Lin Lu, Hui-qin Li, Ji-huang Li, Ai-ju Liu, Guo-qing Zheng

**Affiliations:** Department of Neurology, The Second Affiliated Hospital & Yuying Children's Hospital of Wenzhou Medical University, Wenzhou 325027, China

## Abstract

Sanhua decoction (SHD) is a famous classic Chinese herbal prescription for ischemic stroke, and aquaporin 4 (AQP4) is reported to play a key role in ischemic brain edema. This study aimed to investigate neuroprotection of SHD against focal cerebral ischemia/reperfusion (I/R) injury in rats and explore the hypothesis that AQP4 probably is the target of SHD neuroprotection against I/R rats. Lentiviral-mediated AQP4-siRNA was inducted into adult male Sprague-Dawley rats via intracerebroventricular injection. The focal cerebral ischemia/reperfusion model was established by occluding middle cerebral artery. Neurological examinations were performed according to Longa Scale. Brain water content, was determined by wet and dry weight measurement. Western blot was adopted to test the AQP4 expression in ipsilateral hippocampus. After the treatment, SHD alleviated neurological deficits, reduced brain water content and downregulated the expression of AQP4 at different time points following I/R injury. Furthermore, neurobehavioral function and brain edema after I/R were significantly attenuated via downregulation of AQP4 expression when combined with AQP4-siRNA technology. In conclusion, SHD exerted neuroprotection against focal cerebral I/R injury in rats mainly through a mechanism targeting AQP4.

## 1. Introduction

Stroke is the second leading cause of death worldwide, and the absolute number of stroke patients and the overall global burden of stroke are great and increasing in the past decades [1]. Ischemic stroke is the most common type of stroke, accounting for 60–80% of all types of strokes. However, intravenous rt-PA is the only Food and Drug Administration approved pharmacological therapy for acute ischemic stroke within 4.5 h after stroke onset [2]. Thus, the short therapeutic window [2], low usage rate [3], and safety concerns [4] have prompted a quest for additional therapeutic approaches to acute ischemic stroke.

In China, where the population is 1.4 billion, stroke is already the leading cause of death and adult disability [5]. Fortunately, the important distinction of China's national medical system and Western medicine is that traditional Chinese medicine (TCM) can be responsible for the health care of Chinese people [6]. TCM practitioners use herbal medicines and various mind and body practices, such as acupuncture and tai chi, to treat or prevent health disorders. Sanhua decoction (SHD), a classic Chinese herbal prescription for stroke, was first recorded in* Suwen Bingji Qiyi Baomingji (Plain Questions: Discourse on Mechanism for Preserving Life)* written by Liu Wansu during the periods of Jin and Yuan Dynasties (1115-1368). In modern times, SHD is still used continuously and widely for treatment of stroke [7, 8]. However, the therapeutic mechanism of SHD against acute ischemic stroke still remains unclear.

The water channel protein aquaporin 4 (AQP4), which is the most abundant water channel in mammalian central nervous system, is widely expressed in the ependymal cells and glial membranes bordering the subarachnoidal space, ventricles, and blood vessels [9]. Since it is the structure foundation of water homeostasis and transfer between glial cells, cerebrospinal fluid, and blood vessels, AQP4 plays a key role in maintaining brain water in equilibrium when under physiological conditions, formation of brain edema, and clearance of edema liquid when under pathological conditions [10, 11]. For ischemic stroke, genetic deletion of AQP4 ameliorated brain edema following focal ischemic stroke [12]. Thus, modulating the expression of AQP4 after ischemia stoke is a potential target for the treatment of cerebral ischemic edema.

We proposed a hypothesis that downregulation of AQP4 probably is the target of SHD neuroprotection against I/R injured rats. This study aims to investigate whether SHD could alleviate neurological deficit and provide neuroprotective effects in MCAO induced rat model of focal cerebral I/R injury mainly through a mechanism targeting AQP4.

## 2. Materials and Methods

### 2.1. Experimental Animals

Adult male Sprague-Dawley rats weighing 230–280 g were provided by Shanghai Laboratory Animal Center, CAS (SLACCAS) (NO., SCXK, Shanghai, 2010-0002). Animals were housed in the room with temperature of 21–23°C, relative humidity of 30–70%, and a 12 h light/12 h dark cycle (lights on at 08:00 h). They had free access to common forage and water. All experimental protocols and animal handling procedures were approved by the local ethical committee for animal research and in accordance with the Guide for the Care and Use of Laboratory Animals issued by the National Academy of Sciences, Institute of Laboratory Animal Resources, Commission on Life Sciences, and National Research Council.

### 2.2. Preparation of SHD

SHD is composed of four kinds of CHMs: (A) Radix et Rhizoma Rhei; rhubarb root and rhizome (*dahuang*), the dried root of* Rheum officinale* Baill; (B) Rhizoma et Radix Notopterygii; incised Notopterygium Rhizome and root (*qianghuo*), the dried root of* Notopterygium incisum* Ting ex H. T. Chang; (C) Fructus Aurantii Immaturus; immature orange fruit (*zhishi*), the dried fruitlet of* Citrus aurantium* L.; and (D) Cortex* Magnolia officinalis*; officinal magnolia bark (*houpu*), the dried barks of* Magnolia officinalis* Rehd. et Wils. in the ratio of 4 : 2 : 1.5 : 2 on a dry weight basis, respectively. All the CHMs are recorded in the Chinese Pharmacopoeia. Firstly essential oils are extracted and then decocted with water, extracted thrice, filtered, and concentrated; the raw herbs were made into 1 g·mL^−1^ stock solution. The stock solution was stored at 4°C until use.

### 2.3. Experimental Design

**Figure 1:**
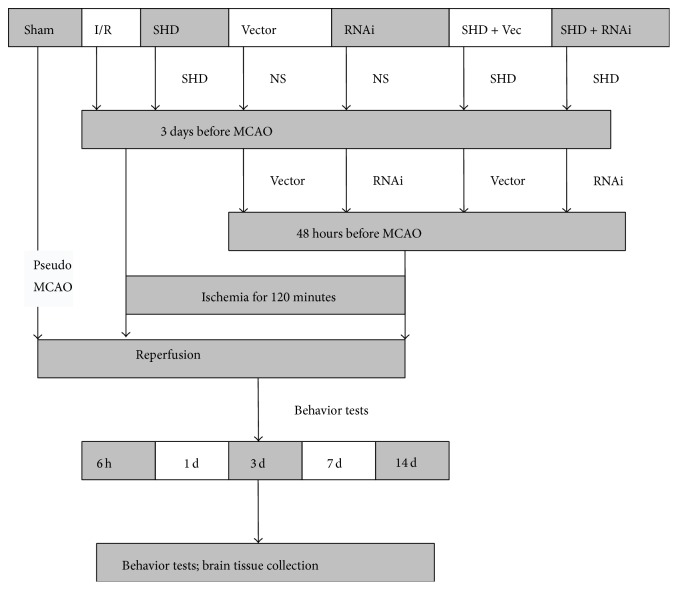
Flow diagram of the experimental protocols. Rats were randomly divided into seven groups. The focal cerebral ischemia/reperfusion model was established in rats by occluding middle cerebral artery (MCAO) with suture for two hours and then extracting the suture. Sham-operated group had the same operation but without suture insertion. In the SHD-treated group, the rats were administered with SHD for 3 days prior to MCAO. In some cases, rats were received by intracerebroventricular injection of either lentiviral-mediated AQP4-siRNA or vehicle 48 hours before ischemic induction. As shown in this figure, neurological functions were observed and brains were collected at indicated time points after reperfusion for brain water content, immunohistochemistry, and western blot analyses. I/R, ischemia/reperfusion group; MCAO, middle cerebral artery occlusion; NS, normal saline; Sham, sham-operated group; SHD, Sanhua decoction-treated group; SHD + RNAi, Sanhua decoction plus AQP4-siRNA-treated group; SHD + Vec, Sanhua decoction plus vector-treated group; RNAi, AQP4-siRNA-treated group; Vector, vector-treated group.

A total of 280 rats were randomly divided into seven groups: sham-operation (Sham) group, cerebral ischemia/reperfusion (I/R) group, Sanhua decoction (SHD) group, Vector group, AQP4-siRNA (RNAi) group, Sanhua decoction + vector (SHD + Vec) group, and Sanhua decoction + AQP4-siRNA (SHD + RNAi) group. Each group was further divided into five subgroups based on different time points. Three days before I/R model establishment, SHD were given to the rats in the three SHD-treated groups by intragastric administration at a dose of 10 g·kg^−1^·d^−1^, once a day till the rats were sacrificed. The other four groups were given the same volume of normal saline. The experimental design of the present study was summarized in a flow diagram (Figure 1).

### 2.4. Focal Cerebral Ischemia/Reperfusion

The focal cerebral ischemia was induced by middle cerebral artery occlusion (MCAO) [13] as described previously in our group [14]. Briefly, the rats were anesthetized by intraperitoneal (i.p.) injection with 10% chloral hydrate at a dose of 350 mg·kg^−1^ after 12 h fasting. After cervical skin preparation and incision, the left common carotid artery (CCA), as well as the external carotid artery (ECA) and internal carotid artery (ICA), was exposed and carefully separated from nerve and tissues. The ECA was clipped and a nylon filament was inserted from it into the lumen of ICA until the blunt tip reached the origin of the MCA. The length of the inserted thread was about 18.5 mm ± 0.5 mm from the CCA bifurcation. Reperfusion was initiated by withdrawal of the occluding filament after 120 min of ischemia. The rats in the Sham group were subjected to the same surgical procedure without suture insertion. Heating pads were used to keep the operated rats warm. A trained operator blinded to the experimental design performed the reversible ischemic injury in animal experiments.

### 2.5. Intracerebroventricular Injection

The lentivirus-mediated aquaporin 4-small interfering RNA (AQP4-siRNA, LV3-Aqp4-rat-543) and its vector were both obtained from GenePharma (Shanghai, China). The target sequence of AQP4-siRNA was 5′-GCTCCTGGTGGAGCTAATAAT-3′. Two days before I/R surgery, AQP4-siRNA was injected into the lateral ventricle of the ischemic side by intracerebroventricular injection: after being anesthetized by intraperitoneal injection of 10% chloral hydrate at a dosage of 350 mg·kg^−1^, the rats were mounted in a stereotactic frame (Huaibei Zhenghua Biological Instrument Equipment Co., LTD) and injected with AQP4-siRNA at the rate of 1 *μ*L·min^−1^ with the volume of 10 *μ*L containing 5 *μ*g AQP4-siRNA using the following stereotaxic coordinates [15]: 0.8 mm posterior to the bregma, 1.5 mm left/right to the midline, and 4.5 mm ventral to the bregma. After the injection, the needle remained in the target location for 5 min to avoid the tracer reflux along the needle tract. The corresponding control groups were given the same volume of vector by intracerebroventricular injection.

### 2.6. Neurological Deficits Scores

Neurological deficits examination was conducted at 6 h, 1 d, 3 d, 7 d, and 14 d after reperfusion by an investigator who was blind to the experiment design according to the five-point scale described previously by Longa et al. [13] as follows: 0 indicated no neurological deficit; 1 mild focal neurological deficit (contralateral forelimb flexion upon lifting the animal tail); 2 moderate focal neurological deficit (circling to the contralateral side when crawling forward); 3 severe focal deficit (falling into the contralateral side when crawling forward); 4 no spontaneous crawling with a depressed level of consciousness or death. Only rats with neurological scores of 1 to 3 were considered successful models and used in the current study.

### 2.7. Calculation of Brain Water Content

Brain water content was measured by the wet/dry method [16]. Briefly, at each indicated time point after reperfusion, the brains were removed after terminal anesthesia and then divided into ipsilateral and contralateral hemispheres. The two hemispheres were weighed, respectively, both before and after drying at 65°C for 48 h. The brain water content was calculated using the formula: brain  water  content = (wet  weight − dry  weight)/wet  weight × 100%.

### 2.8. Western Blotting Analysis

Western blot was adopted to evaluate the expression level of AQP4 in ipsilateral hippocampus. Whole cells of samples were lysed with RIPA lysis buffer (P0013B, Beyotime Institute of Biotechnology, Jiangsu, China) and the protein was determined with a BCA kit (Beyotime Institute of Biotechnology, Jiangsu, China). Equal amount of protein was separated by 10% Tris-glycine SDS-PAGE polyacrylamide gel and transferred to polyvinylidene fluoride membranes (Invitrogen, USA). After blocking for 1.5 h with a 5% solution of skim milk (232100, BD-Difco, USA), the membranes were incubated with the primary antibodies polyclonal rabbit anti-AQP4 (1 : 1000; ab46182, Abcam, USA) or polyclonal rabbit anti-GAPDH (1 : 1000; AP0063, Bioworld Technology, USA) at 4°C overnight, followed by the horseradish peroxidase- (HRP-) linked anti-goat antibody (1 : 10000; Boyun Biotech, Shanghai, China) for 30–60 min at room temperature. After washing with Tris-buffered saline with 0.1% Tween-20 (Beyotime Institute of Biotechnology, Jiangsu, China), the relative intensity of protein signals was normalized to the corresponding GAPDH intensity and was quantified by densitometric analysis with the use of Quantity One software (Bio-Rad Laboratories, USA).

### 2.9. Statistical Analysis

All experimental data were presented as mean ± standard deviation. Neurologic deficit score data were analyzed using a nonparametric Mann-Whitney* U* test. Paired* t*-test was used for the significant difference of brain water content between ipsilateral and contralateral hemispheres. Comparisons between multiple groups were done by one-way analysis of variance (ANOVA) followed by LSD-*t*-test. Statistical analyses were performed with SPSS 13.0 for Windows (Chicago, IL, USA). Statistical significance was set at *P* < 0.05.

## 3. Results

### 3.1. SHD Alleviated Neurological Deficits

**Figure 2:**
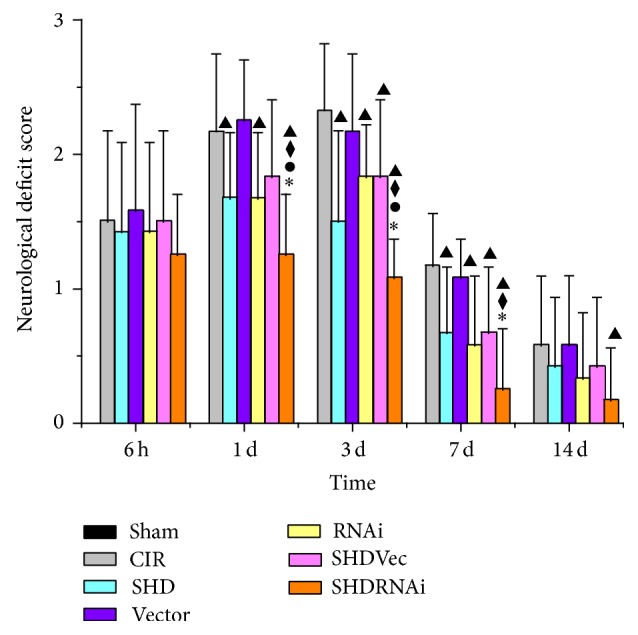
Neurological deficits after ischemia/reperfusion in rats ($\overset{-}{x}\pm s,n=8$). ^▲^ *P* < 0.05, compared with CIR group; ^◆^ *P* < 0.05, compared with SHD group; ^∗^ *P* < 0.05, compared with SHD + Vec group; ^●^ *P* < 0.05, compared with RNAi group. CIR, cerebral ischemia/reperfusion group; Sham, sham-operated group; SHD, Sanhua decoction-treated group; SHD + RNAi, Sanhua decoction plus AQP4-siRNA-treated group; SHD + Vec, Sanhua decoction plus vector-treated group; RNAi, AQP4-siRNA-treated group; Vector, vector-treated group.

Neurological scores increased at 6 h after ischemia/reperfusion and peaked at 3 d and then descended gradually but remained higher than normal at 14 d (Figure 2). Compared with Sham group, I/R group showed significant differences at the time points of 6 h, 1 d, 3 d, and 7 d (*P* < 0.05). Compared with I/R group, SHD + Vec group has significant differences at 3 d and 7 d (*P* < 0.05), SHD group and RNAi group both showed significances at 1 d, 3 d, and 7 d (*P* < 0.05), and SHD + RNAi group presented statistical differences at 1 d, 3 d, 7 d, and 14 d (*P* < 0.05). Compared with SHD group or SHD + Vec group, SHD + RNAi group had significant differences at 1 d, 3 d, and 7 d after I/R (*P* < 0.05). There were also statistical differences between RNAi group and SHD + RNAi group at the time points of 1 d and 3 d (*P* < 0.05). However, there was no significant difference between SHD group and RNAi group, Vector group and I/R group, SHD group and SHD + Vec group, and RNAi group and SHD + Vec group, at all the time points of 6 h, 1 d, 3 d, and 7 d (*P* > 0.05). Thus, SHD can alleviate neurological deficits and its effect was amplified by combination of AQP4-siRNA.

### 3.2. SHD Reduced Brain Water Content

**Figure 3:**
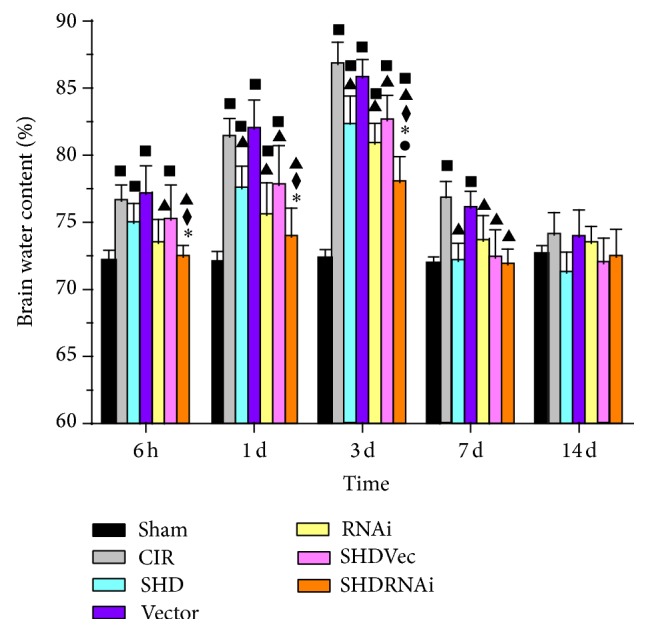
Brain water content of the ipsilateral side after ischemia/reperfusion in rats ($\overset{-}{x}\pm s,n=4$). ^■^ *P* < 0.05, compared with Sham group; ^▲^ *P* < 0.05, compared with CIR group; ^◆^ *P* < 0.05, compared with SHD group; ^∗^ *P* < 0.05, compared with SHD + Vec group; ^●^ *P* < 0.05, compared with RNAi group. CIR, cerebral ischemia/reperfusion group; Sham, sham-operated group; SHD, Sanhua decoction-treated group; SHD + RNAi, Sanhua decoction plus AQP4-siRNA-treated group; SHD + Vec, Sanhua decoction plus vector-treated group; RNAi, AQP4-siRNA-treated group; Vector, vector-treated group.

Brain water content of contralateral hemisphere had no difference between groups (*P* > 0.05). In ipsilateral hemisphere, it increased at 6 h after I/R and peaked at 3 d and then descended gradually but remained higher than normal at 14 d (Figure 3). Compared with Sham group, I/R group and Vector group both had significant differences at the time points of 6 h, 1 d, 3 d, and 7 d (*P* < 0.05), RNAi group had significant differences at 1 d and 3 d (*P* < 0.05), and SHD + RNAi group had significant differences at 3 d (*P* < 0.05). Compared with I/R group, SHD group and SHD + Vec group both had differences at 1 d, 3 d, and 7 d (*P* < 0.05), and RNAi group and SHD + RNAi group both showed significances at 6 h, 1 d, 3 d, and 7 d (*P* < 0.05). Compared with SHD group or SHD + Vec group, SHD + RNAi group had statistical differences at 6 h, 1 d, and 3 d (*P* < 0.05). There was statistical significance between RNAi group and SHD + RNAi group at the time point of 3 d (*P* < 0.05) (Figure 3). Thus, SHD can reduce brain water content and it was remarkable in combination with AQP4-siRNA.

### 3.3. SHD Downregulated the Expression of AQP4

**Figure 4:**
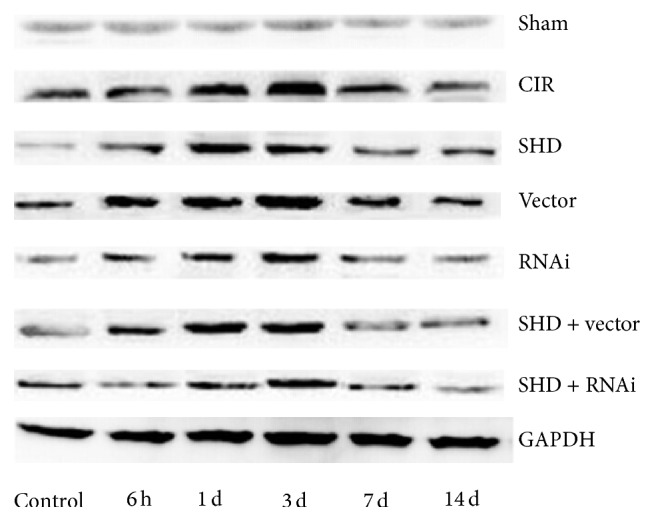
Aquaporin 4 (AQP4) expression in the ipsilateral hippocampus by western blot. CIR, cerebral ischemia/reperfusion group; Sham, sham-operated group; SHD, Sanhua decoction-treated group; SHD + RNAi, Sanhua decoction plus AQP4-siRNA-treated group; SHD + Vec, Sanhua decoction plus vector-treated group; RNAi, AQP4-siRNA-treated group; Vector, vector-treated group.

**Figure 5:**
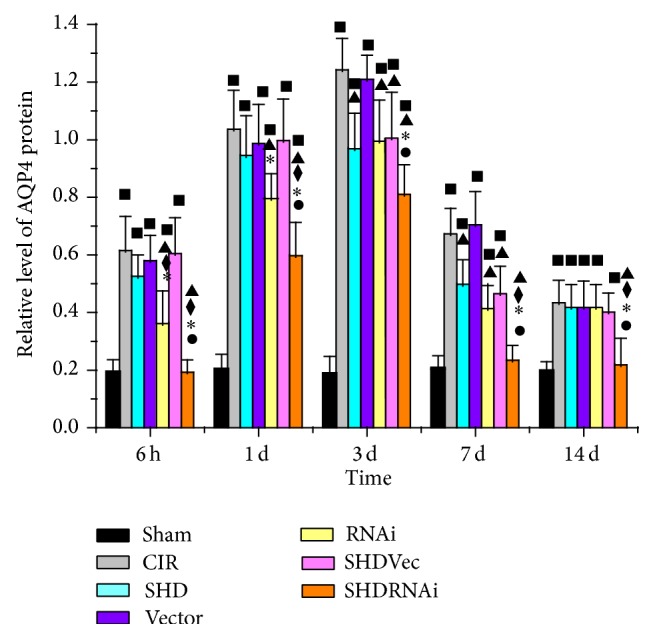
Western blot analyses of AQP4 expression in the ipsilateral hippocampus after ischemia/reperfusion in rats ($\overset{-}{x}\pm s,n=4$). ^■^ *P* < 0.05, compared with Sham group; ^▲^ *P* < 0.05, compared with CIR group; ^◆^ *P* < 0.05, compared with SHD group; ^∗^ *P* < 0.05, compared with SHD + Vec group; ^●^ *P* < 0.05, compared with RNAi group. CIR, cerebral ischemia/reperfusion group; Sham, sham-operated group; SHD, Sanhua decoction-treated group; SHD + RNAi, Sanhua decoction plus AQP4-siRNA-treated group; SHD + Vec, Sanhua decoction plus vector-treated group; RNAi, AQP4-siRNA-treated group; Vector, vector-treated group.

Expression of AQP4 was detected by WB analyses. The expression of AQP4 in the ipsilateral hippocampus increased at 6 h after ischemia/reperfusion and peaked at 3 d and then descended gradually but remained higher than normal at 14 d (Figure 4). Compared with Sham group, SHD + RNAi group had statistical significances at 1 d and 3 d after I/R (*P* < 0.05), and the other 5 groups all showed differences at each time point after I/R (*P* < 0.05). Compared with I/R group, SHD group and SHD + Vec group both had significant differences at the time points of 3 d and 7 d (*P* < 0.05), RNAi group had differences at 6 h, 1 d, 3 d, and 7 d (*P* < 0.05), and SHD + RNAi group showed significance at each time point (*P* < 0.05). SHD + RNAi group showed significant differences at each time point after I/R when compared with SHD group, SHD + Vec group, or RNAi group (*P* < 0.05) (Figure 5). Thus, SHD can downregulate AQP4 expression in rat brain tissue and it was more notable when combined with AQP4-siRNA.

## 4. Discussion

In the present study, we demonstrated that SHD treatment could alleviate neurological deficit, reduce the brain water content, and decline AQP4 expression in peripheral ischemic lesions after I/R. Furthermore, neurobehavioral function and brain edema after I/R were significantly attenuated via downregulation of AQP4 expression when combined with AQP4-siRNA technology.

Transient suture-occluded MCAO method in rats is the most common and preferred focal I/R injury model for imitating human ischemic stroke [14]. Evaluation of neurological function impairment after cerebral ischemia according to Longa test with 5-point scale, which is highly recommended worldwide, was set up by the same author who created transient MCAO with the suture [13]. In the present study, this neurologic grading scale was applied to assess the degree of neurological deficits following suture-occluded MCAO for 2 h in rats. We found that SHD treatment notably reduced neurological deficit scores in rats with MCAO. More remarkable improvement for neurological deficits was shown when combining SHD with AQP4-siRNA technology. These findings suggest that SHD treatment promotes behavioral functional recovery after ischemic stroke. With AQP4-siRNA technology, SHD had stronger and more stable effect on the functional recovery.

Brain edema is detrimental because of its increasing volume occupied consequences having a swelling effect on adjacent tissues, and these effects were magnified by the fixed volume of the skull. The tissues were compromised with capillary inflow, leading to ischemia and edema formation in reverse, causing a secondary injury in brain [17]. It is broadly acknowledged that brain water content is practicable for evaluating brain edema using dry and wet method. In the present study, we applied this weighing method with Bolliot formula to assess the extent of brain edema after cerebral ischemia. The data showed that brain water content increased immediately after MCAO in rats, SHD treatment could effectively inhibit the increase of water content, and the combined effect of SHD treatment with AQP4-siRNA had promoted decrease of water content more significantly. These results suggested that brain edema rapidly formed in the acute stage of I/R injury, which was obviously alleviated by SHD treatment and more prominently attenuated by SHD treatment integrated with AQP4-siRNA.

Although bumetanide [18], acetazolamide [19], and erythropoietin [20] were proposed to decrease AQP4 expression, there are no specific therapeutic blockers to inhibit the AQP4 channel at present and such agents are vital to evaluating the role and treatment of edema. In the present study, we investigated the effect of SHD on expression levels of AQP4 in hippocampus of I/R injured rats. Basically consistent with the result of brain water content, the data showed that AQP4 was upregulated and markedly expressed in the ipsilateral hemisphere after I/R, SHD treatment significantly inhibited the expression of AQP4, and such downregulated expression was magnified by the combination of SHD with AQP4-siRNA. Analyses of these results revealed that AQP4 was involved in the formation of brain edema after cerebral ischemia, and SHD treatment reduced AQP4 expression corresponding to its protective effect against brain edema. With the AQP4-siRNA technology, it provided further evidence that SHD treatment likely exerts neuroprotective effects following cerebral ischemia mainly via blocking AQP4 channel [21].

SiRNA, a short 21–23-nucleotide double-stranded RNA molecule that mediates sequence-specific gene silencing, was performed in several animal studies as potential therapeutics in acute brain injury, such as brain ischemia, brain hemorrhage, and traumatic brain injury [22–24]. Applying siRNA to stroke patient in the near future, a very promising method of therapy, is expected to lead to a global breakthrough in improving functional recovery [21]. Direct transfection of chemically synthetic siRNA and intracellular generation of siRNA from plasmid or viral vectors which drive expression of the precursor shRNA can both achieve RNAi that has been widely used in gene research and therapy. Because of active infection on dividing as well as resting and differentiated cells, viral DNA incorporated in the host genome, and relatively low immunogenicity, lentiviruses are particularly suited for long-term shRNA expression and gene silencing [25]. The present experimental results showed that AQP4-siRNA with intracerebroventricular injection could effectively reduce neurologic deficit scores, as well as brain water content in MCAO rats, and such benefits were amplified by the combination of siRNA with SHD treatment. The amelioration of neurological functional recovery and reduction of brain edema with AQP4-siRNA after brain injury was consistent with that in the study by Fukuda et al. [26]. Thus, the neuroprotection effect of SHD against I/R injury was mainly carried out by AQP4 blocking. However, the specific pathway of downregulating AQP4 expression involved in SHD deserves further exploration. In addition, vascular protection is the key for development of therapeutic agents for stroke [27]. Thus, whether SHD impacts the vascular factors related to ischemic brain damage is worthy of further study.

In conclusion, the present study demonstrated that SHD can improve neurological function deficits and reduce brain water content after focal cerebral ischemia. The mechanism of this neuroprotection by SHD after cerebral I/R mainly targets downregulation of AQP4 expression.
